# Neck circumference as an independent predictor for NAFLD among postmenopausal women with normal body mass index

**DOI:** 10.1186/s12986-021-00562-3

**Published:** 2021-03-17

**Authors:** Jie Shi, Zixuan Wang, Weiwei Zhang, Yixin Niu, Ning Lin, Xiaoyong Li, Hongmei Zhang, Guang Ning, Jiangao Fan, Li Qin, Qing Su, Zhen Yang

**Affiliations:** 1 Department of Endocrinology, Xinhua Hospital, Shanghai Jiao Tong University School of Medicine, Shanghai, China; 2 Department of Gastroenterology, Shanghai Key Laboratory of Children’s Digestion and Nutrition, Xinhua Hospital, Shanghai Jiao Tong University School of Medicine, Shanghai, China; 3 Shanghai National Clinical Research Center for Endocrine and Metabolic Diseases, Key Laboratory for Endocrine and Metabolic Diseases of the National Health Commission of the PR China, Shanghai Institute of Endocrine and Metabolic Diseases, Ruijin Hospital, Shanghai Jiao Tong University School of Medicine, Shanghai, China

**Keywords:** Neck circumference, Nonalcoholic fatty liver disease, Postmenopausal women, Normal body mass index

## Abstract

**Background:**

Neck circumference, a proxy for upper-body subcutaneous fat, is a unique and pathogenic fat depot that confers additional metabolic risk. The purpose of present study was to determine whether neck circumference associates with nonalcoholic fatty liver disease (NAFLD) in postmenopausal women with normal body mass index.

**Methods:**

A cross-sectional survey (n = 2492) and a 3.1-year follow-up investigation (n = 1354) were conducted among Chinese postmenopausal women with normal BMI (18.5 to < 25 kg/m^2^). Neck circumference was measured horizontally at the lower margin of the laryngeal prominence.

**Results:**

In the cross-sectional analysis, large neck circumference was associated with the presence of NAFLD (odds ratio 2.28; 95% CI 1.74–2.98; highest tertile versus lowest tertile) after adjustment for confounding factors. Among 1354 subjects without the NAFLD at baseline, 429 (31.7%) incident NAFLD cases occurred at 3.1 years. Neck circumference was positively associated with triglycerides, homeostasis model assessment of insulin resistance, C-reactive protein, and negatively associated with high-density lipoprotein cholesterol and adiponectin. Individuals with large baseline neck circumference had a significantly higher risk of NAFLD than those with small neck circumference. The multivariable adjusted hazard ratio was 1.42 (95% CI 1.15–1.97; *p* for trend = 0.004) for the highest versus the lowest tertile of neck circumference, and was 1.22 (95% CI 1.10–1.41; *p* = 0.006) per 1-standard deviation increment in neck circumference.

**Conclusions:**

Among postmenopausal women with normal BMI, relatively large neck circumference levels are associated with an increased risk of NAFLD.

**Supplementary Information:**

The online version contains supplementary material available at 10.1186/s12986-021-00562-3.

## Introduction

Nonalcoholic fatty liver disease (NAFLD) is characterized by excessive fat accumulation in the liver with the absence of alcohol and other liver diseases. It is well established that adiposity is a major contributor to NAFLD. Data from epidemiological studies revealed that NAFLD occurrence in up to 70% of overweight adults worldwide [1] and in 90% of morbidly obese adults globally [2]. Nonetheless, a remarkable proportion of individuals having NAFLD with a relatively normal body mass index (BMI), a condition termed as ‘‘non-obese” NAFLD. The prevalence of NAFLD was around 10% in nonobese adults in Western countries, and about 8–19% of Asians with BMI less than 25 kg/ m^2^ are also found to have NAFLD [3]. Given this trend, a reliable predictor of NAFLD in nonobese people is essential for its prevention and management.

As we best known, the larger the BMI, the higher the metabolic risk. However, individuals within the same BMI status could have considerable differences in the amount and distribution of regional fat depots, leading to variable metabolic risks [4, 5]. Numerous researches highlight the metabolic risk conferred by specific patterns of fat distribution, particularly upper body adiposity [6]. Notably, several studies have recently shown that upper-body subcutaneous adipose tissue, estimated by neck circumference, is a unique fat depot that confer additional metabolic risks beyond generalized and abdominal adiposity [7, 8]. It is reported that neck circumference levels are significantly associated with hyperlipidemia, impaired glucose homeostasis, and insulin resistance, especially in women [7]. All of these supported the investigation of a possible association of neck circumference with NAFLD.

Due, in part, to a transfer from subcutaneous to abdominal visceral fat, women after menopause are prone to metabolic changes [9]. Such metabolic alterations are associated with increased NAFLD risk among normal BMI populations. However, studies that assess neck fat accumulation, as a proxy for upper-body subcutaneous fat, and its relationship with NAFLD among postmenopausal women with normal BMI are still lacking. In the current study, we evaluated the relationship between neck circumference and NAFLD among postmenopausal women with normal BMI.

## Participants and methods

### Study subjects

**Fig. 1:**
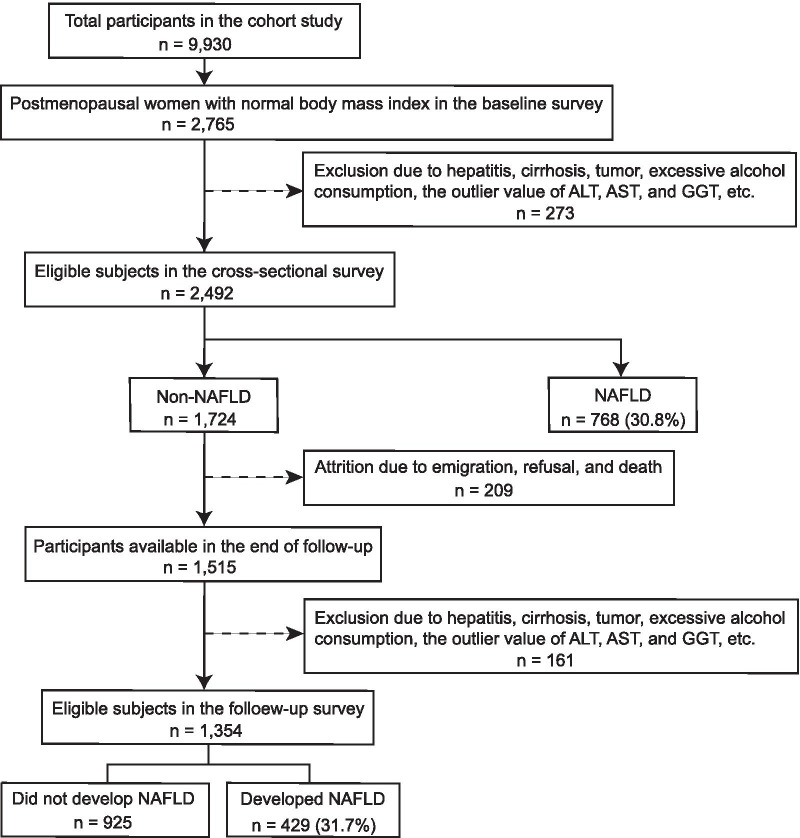
Overview of the study design

Participants were recruited from the China Cardiometabolic Disease and Cancer Cohort (4C) Study, a nationwide prospective cohort study investigating the associations of metabolic factors with specific clinical outcomes, including diabetes, cardiovascular disease, cancer, and all-cause mortality [10, 11]. The data presented in this study are based on the subsamples from the Chongming District in Shanghai, China. From May to November 2011, a total of 2765 postmenopausal women with normal BMI of Chinese origins were enrolled in the study. From June to December 2014, the subjects were invited for follow-up assessments. The cross-sectional survey included 2492 subjects, and the follow-up investigation included 1354 individuals without NAFLD at baseline (Fig. 1). Subjects with the following conditions were excluded from this study: virus hepatitis, autoimmune hepatitis, drug-induced liver disease, current drinkers, ex-drinkers, presence of tumor, biliary obstructive diseases, thyroid dysfunction, total parenteral nutrition, Wilson’s disease, severe renal insufficiency, significant hematologic disorders, and current treatment with systemic corticosteroids.

The study protocol was approved by the Ethics Committee of Xinhua Hospital Affiliated to Shanghai Jiao Tong University School of Medicine. Written informed consent was obtained from all participants.

### Clinical diagnosis of NAFLD

Guidelines for the diagnosis of NAFLD proposed by the Asia–Pacific Working Party were used [12]. NAFLD was clinically defined as manifestations of B-mode ultrasonography, after the exclusion of the habit of drinking and the history of specific diseases that could lead to fatty liver. Abdominal ultrasonography was performed by experienced ultrasonographers who were blinded to clinical presentation and laboratory data. Hepatic steatosis was defined as a diffuse increase of fine echoes in the liver parenchyma compared with that in the kidney or spleen parenchyma based on standard criteria.

### Anthropometric and biochemical measurements

Neck circumference was measured horizontally at the lower margin of the laryngeal prominence (Adam’s apple), with head erect and eyes facing forward. BMI was calculated as the weight in kilograms divided by the square of the height in meters. Waist circumference was measured at the midpoint between the inferior costal margin and the superior border of the iliac crest on the midaxillary line.

Serum total cholesterol, high-density lipoprotein cholesterol (HDL-C), low-density lipoprotein cholesterol, triglycerides, alanine aminotransferase, aspartate aminotransferase, and γ-glutamyltranspeptidase were measured on an autoanalyzer (Hitachi 7080; Tokyo, Japan). Venous plasma glucose level was determined by glucose oxidase method (ADVIA-1650 Chemistry System, Bayer, Leverkusen, Germany) and hemoglobin A1c was measured by high-performance liquid chromatography (BIO-RAD, D10, CA). Fasting insulin was measured by RIA (Linco Research, St. Charles, MO). The homeostasis model assessment-insulin resistance (HOMA-IR) was used to assess insulin resistance, which was calculated using the following equation: HOMA-IR = insulin (uU/mL) * glucose (mmol/L)/22.5 [13]. Serum C-reactive protein (CRP) and adiponectin levels were quantified using the enzyme-linked immunosorbent assay (ELISA) kits (DY1707, DY1065; R&D Systems, Minneapolis, MN).

### Statistical analysis

The continuous variables with normal distribution are expressed as means ± SDs. The continuous variables with skewed distribution are shown as medians (interquartile range) and log-transformed to approximate normality before analysis. Categorical variables are reported as frequencies (%). For comparisons between groups, we conducted an independent-samples Student t-test for normally distributed variables and a Mann–Whitney U test for variables with highly skewed distributions. The Chi-squared test was performed to compare categorical variables. The correlation coefficients between neck circumference and metabolic parameters were calculated using the Pearson correlation analysis and Partial correlation analysis after adjustment for age, smoking, physical activity, educational attainment, body mass index, and waist circumference. Logistic regression analyses were used to assess the relationship between neck circumference and the prevalence of NAFLD in the cross-sectional survey. Model 1 was adjusted for age, smoking status, physical activity, and educational attainment. Model 2 was further adjusted for BMI and waist circumference. Model 3 was further adjusted for HOMA-IR, CRP, and adiponectin. Model 4 was further adjusted for model 3 variables and fasting glucose, post-loading plasma glucose, systolic blood pressure, diastolic blood pressure. Model 5 was further adjusted for lipid profiles and liver enzymes.

Multivariate Cox regression analyses were run to evaluate the potential association between neck circumference and the incidence of NAFLD. Covariates were selected on the basis of biologic interest, well-established risk factors for NAFLD, or associated exposures and outcomes. Variables showing *p* < 0.05 in the univariable regression were entered into the multivariable model. Multivariable adjusted models were used to explore the independent effect of neck circumference on the incidence of NAFLD. We also used restricted cubic splines with five knots at percentiles 5%, 35%, 50%, 65%, and 95% of the distribution to flexibly model to assess the association of neck circumference on a continuous scale and the incidence of NAFLD. Hazard ratios (HRs) and 95% confidence intervals (CIs) for the relationship between neck circumference and the incidence of NAFLD were generated with the Cox regression models. The surface fitting based on the least square method was performed to assess joint effect of neck circumference with BMI, waist circumference, and HOMA-IR on NAFLD after adjustment for age, smoking status, physical activity, educational attainment, BMI, waist circumference, HOMA-IR, CRP, adiponectin, fasting plasma glucose, post-loading plasma glucose, systolic blood pressure, diastolic blood pressure, lipid profiles, and liver enzymes.

Several risk factors may affect the association between neck circumference and incident NAFLD, particularly age, BMI, waist circumference, CRP, diabetes status, and physical activity. Consequently, we conducted a subgroup analysis to examine whether the relationship between neck circumference and the risk of incident NAFLD was robust in the presence of potential confounders. Subgroups were stratified by age < 65 years versus age ≥ 65 years, BMI < 23 kg/m^2^ versus BMI ≥ 23 kg/m^2^, waist circumference < 80 cm versus waist circumference ≥ 80 cm, CRP < 3.0 mg/L versus CRP ≥ 3.0 mg/L, without diabetes versus with diabetes, and low physical activity versus moderate physical activity versus high physical activity.

A 2-tailed *p* < 0.05 was considered statistically significant. All analyses were performed using R version 4.0.2 and SPSS software version 25.0.

## Results

### Association between neck circumference and the presence of NAFLD in the cross-sectional survey

The clinical characteristics of the study subjects are shown in Table 1. Subjects with NAFLD had a higher BMI, waist circumference, triglycerides levels, and CRP levels, and a lower HDL-C and adiponectin levels. Larger neck circumference was observed in individuals with NAFLD than those without NAFLD (33.4 ± 2.6 cm vs. 32.0 ± 2.4 cm, *p* < 0.001). When stratified by tertiles of neck circumference, the prevalence of NAFLD raised sharply from the lowest tertile to the highest tertile group (15.9%, 29.7%, 47.5%, respectively). According to logistic regression analyses, large neck circumference was associated with the presence of NAFLD (odds ratio, 2.28; 95% CI, 1.74–2.98; highest versus lowest tertile, *p* < 0.001) after adjustment for age, smoking status, physical activity, educational attainment, BMI, waist circumference, HOMA-IR, CRP, adiponectin, fasting plasma glucose, post-loading plasma glucose, systolic blood pressure, diastolic blood pressure, lipid profiles, and liver enzymes (Table 2).

**Table 1:** Characteristics of subjects according to the presence or absence of the nonalcoholic fatty liver diseases at baseline (n = 2492)

Variables	Non-NAFLD (n = 1724)	NAFLD (n = 768)	*p* value
Age (years)	58.7 ± 5.6	58.8 ± 5.3	0.62
Current smoker, n (%)	78 (4.5)	36 (4.7)	0.88
Educational attainment, n (%)			0.48
0–6	448 (26.0)	202 (26.3)	
7–9	781 (45.3)	363 (47.3)	
≥ 10	495 (28.7)	203 (26.4)	
Physical activity, n (%)			0.52
Low	1258 (73.0)	577 (75.1)	
Moderate	343 (19.9)	139 (18.1)	
High	123 (7.1)	52 (6.8)	
BMI (kg/m^2^)	22.22 ± 1.61	23.27 ± 1.40	< 0.001
Waist circumference (cm)	78.2 ± 7.1	82.6 ± 6.6	< 0.001
SBP (mmHg)	127.77 ± 18.13	131.27 ± 18.57	< 0.001
DBP (mmHg)	77.15 ± 9.73	79.47 ± 9.66	< 0.001
FPG (mmol/L)	6.00 ± 1.36	6.68 ± 2.06	< 0.001
PPG (mmol/L)	7.96 ± 3.25	9.87 ± 4.47	< 0.001
HbA_1c_ (%)	5.90 ± 0.84	6.30 ± 1.22	< 0.001
HOMA-IR	1.49 (1.16–1.93)	2.29 (1.65–2.97)	< 0.001
HDL-C (mmol/L)	1.33 ± 0.32	1.19 ± 0.30	< 0.001
LDL-C (mmol/L)	2.67 ± 0.76	2.71 ± 0.82	0.174
Total cholesterol (mmol/L)	4.74 ± 1.00	4.86 ± 1.06	0.011
Triglycerides (mmol/L)	1.18 (0.89–1.60)	1.71 (1.21–2.50)	< 0.001
ALT (U/L)	12 (9–16)	15 (11–23)	< 0.001
AST (U/L)	18 (15–22)	19 (16–24)	0.004
GGT (U/L)	15 (11–20)	20 (14–31)	< 0.001
CRP (mg/L)	1.09 (0.58–2.01)	2.04 (1.10–3.16)	< 0.001
Adiponectin (mg/L)	3.81 ± 1.17	3.19 ± 0.97	< 0.001
Neck circumference (cm)	32.0 ± 2.4	33.4 ± 2.6	< 0.001

ALT, alanine aminotransferase; AST, aspartate transaminase; BMI, body mass index; CRP, C-reactive protein; DBP, diastolic blood pressure; FPG, fasting plasma glucose; GGT, γ-glutamyltransferase; HbA_1c_, glycated hemoglobin; HDL-C, high-density lipoprotein cholesterol; HOMA-IR, homeostasis model assessment-insulin resistance; LDL-C, low-density lipoprotein cholesterol; PPG, postprandial plasma glucose; SBP, systolic blood pressure

**Table 2:** Presence of nonalcoholic fatty liver diseases in relation to neck circumference tertiles (n = 2492)

	Neck circumference			*p* for trend
	Tertile 1 (n = 830)	Tertile 2 (n = 831)	Tertile 3 (n = 831)	
Prevalence of NAFLD (%)	132 (15.9)	247 (29.7)	395 (47.5)	< 0.001
Model 1 OR (95% CI)	1.00	2.24 (1.77–2.84)	4.79 (3.80–6.04)	< 0.001
Model 2 OR (95% CI)	1.00	1.80 (1.41–2.30)	3.52 (2.76–4.48)	< 0.001
Model 3 OR (95% CI)	1.00	1.70 (1.33–2.18)	3.05 (2.38–3.92)	< 0.001
Model 4 OR (95% CI)	1.00	1.64 (1.28–2.10)	2.84 (2.21–3.65)	< 0.001
Model 5 OR (95% CI)	1.00	1.42 (1.09–1.85)	2.28 (1.74–2.98)	< 0.001

Model 1 was adjusted for age, smoking status, physical activity, and educational attainment. Model 2 was further adjusted for BMI and waist circumference. Model 3 was further adjusted for HOMA-IR, CRP, and adiponectin. Model 4 was further adjusted for model 3 variables and fasting glucose, post-loading plasma glucose, systolic blood pressure, diastolic blood pressure. Model 5 was further adjusted for lipid profiles and liver enzymes

### Association between baseline neck circumference and the incidence of NAFLD in the follow-up investigation

During a 3.1-year follow-up period, 429 participants developed NAFLD. The clinical characteristics of the study subjects are presented in Table 3. No statistically significant difference in baseline characteristics was found between those retained and those lost to follow-up (Additional file 1: Table S1). According to Pearson correlations analyses, the neck circumference was positively correlated with BMI, waist circumference, triglycerides, HOMA-IR, and CRP. In contrast, neck circumference was negatively correlated with HDL-C and adiponectin (Table 4). The associations were still significant even after adjustment for age, smoking, physical activity, educational attainment, BMI, and waist circumference (*p* < 0.05).

**Table 3:** Baseline characteristics of subjects with or without development of nonalcoholic fatty liver diseases at 3.1 years (n = 1354)

Variables	Non-NAFLD (n = 925)	NAFLD (n = 429)	*p* value
Age (years)	58.6 ± 5.7	58.7 ± 5.5	0.678
Current smoker, n (%)	50 (5.4)	19 (4.4)	0.34
Educational attainment, n (%)			0.26
0–6	235 (25.4)	120 (28.0)	
7–9	419 (45.3)	201 (46.9)	
≥ 10	271 (29.3)	108 (25.2)	
Physical activity, n (%)			0.95
Low	672 (72.6)	308 (71.8)	
Moderate	189 (20.4)	90 (21.0)	
High	64 (6.9)	31 (7.2)	
BMI (kg/m^2^)	21.95 ± 1.61	22.79 ± 1.48	< 0.001
Waist circumference (cm)	77.3 ± 6.7	80.5 ± 6.1	< 0.001
SBP (mmHg)	126.91 ± 17.89	129.38 ± 18.29	0.023
DBP (mmHg)	76.95 ± 9.66	78.21 ± 9.70	0.031
FPG (mmol/L)	5.88 ± 1.20	6.20 ± 1.55	< 0.001
PPG (mmol/L)	7.61 ± 2.95	8.48 ± 3.42	< 0.001
HbA_1c_ (%)	5.84 ± 0.71	5.93 ± 0.96	0.104
HOMA-IR	1.36 (1.00–1.79)	1.72 (1.36–2.41)	< 0.001
HDL-C (mmol/L)	1.36 ± 0.34	1.28 ± 0.31	< 0.001
LDL-C (mmol/L)	2.67 ± 0.77	2.70 ± 0.76	0.552
Total cholesterol (mmol/L)	4.75 ± 1.03	4.80 ± 1.00	0.464
Triglycerides (mmol/L)	1.14 (0.86–1.51)	1.40 (0.99–1.96)	< 0.001
ALT (U/L)	11 (8–15)	13 (10–16)	< 0.001
AST (U/L)	19 (15–23)	19 (15–22)	0.781
GGT (U/L)	14 (11–19)	16 (12–23)	< 0.001
CRP (mg/L)	1.04 (0.57–1.95)	1.28 (0.68–2.40)	< 0.001
Adiponectin (mg/L)	3.98 ± 1.16	3.46 ± 1.13	< 0.001
Neck circumference (cm)	31.6 ± 2.2	32.4 ± 2.2	< 0.001

ALT, alanine aminotransferase; AST, aspartate transaminase; BMI, body mass index; CRP, C-reactive protein; DBP, diastolic blood pressure; FPG, fasting plasma glucose; GGT, γ-glutamyltransferase; HbA1c, glycated hemoglobin; HDL-C, high-density lipoprotein cholesterol; HOMA-IR, homeostasis model assessment-insulin resistance; LDL-C, low-density lipoprotein cholesterol; PPG, postprandial plasma glucose; SBP, systolic blood pressure

**Table 4:** Correlations between baseline neck circumference and metabolic characteristics

Variables	Neck circumference			
	Univariate		Multivariate	
	Beta	*p* value	Beta	*p* value
Body mass index	0.230	< 0.001	–	–
Waist circumference	0.313	< 0.001	–	–
HDL-C	− 0.144	< 0.001	-0.12	< 0.001
Triglycerides	0.104	< 0.001	0.11	< 0.001
HOMA-IR	0.128	< 0.001	0.18	< 0.001
CRP	0.103	0.002	0.10	0.003
Adiponectin	-0.110	< 0.001	-0.09	0.007

Multivariable model was adjusted for age, smoking, physical activity, educational attainment, body mass index, and waist circumference. CRP, C-reactive protein; HDL-C, high-density lipoprotein cholesterol; HOMA-IR, homeostasis model assessment-insulin resistance

**Fig. 2:**
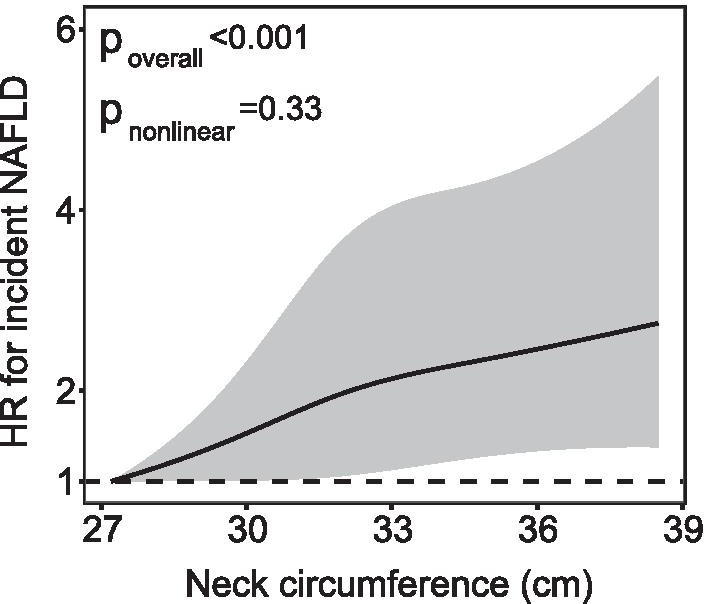
Neck circumference on a continuous scale and risk of incident nonalcoholic fatty liver diseases. Data were fit by a Cox regression model based on restricted cubic splines with 5 knots at the 5th, 35th, 50th, 65th, and 95th percentiles. The solid line represents the hazard ratio and the gray area represents the 95% confidence interval. Model was adjusted for age, smoking status, physical activity, educational attainment, BMI, waist circumference, HOMA-IR, CRP, adiponectin, fasting plasma glucose, post-loading plasma glucose, systolic blood pressure, diastolic blood pressure, lipid profiles, and liver enzymes

We used Cox regression analysis with the lowest neck circumference tertile group as a reference to assess the relationship between neck circumference and the risk of incident NAFLD. As presented in Table 5, the HRs for incident NAFLD was higher with increasing neck circumference tertiles. In the highest neck circumference tertile, the HR was 2.16 (95% CI 1.60–2.92; *p* for trend < 0.001) for incident NAFLD after adjusting for age, smoking, physical activity, and educational attainment (model 1). Interestingly, further adjustment for BMI and waist circumference (model 2) only slightly reduced the magnitude of the HRs for incident NAFLD. Furthermore, there were still statistically significant (HR 1.42; 95% CI 1.15–1.97; *p* for trend = 0.004) by additional adjustment for HOMA-IR, CRP, adiponectin, glucose parameters, systolic blood pressure, diastolic blood pressure, lipid profiles, and liver enzymes (model 5). When neck circumference was considered as a continuous variable, the overall HR (95% CI) of having NAFLD was 1.22 (1.10–1.41) per 1-SD increment of neck circumference. A positive linear circumference-response relationship was evident in the cubic spline regression model (Fig. 2, p for nonlinearity > 0.1).

**Table 5:** Incidence of NAFLD in relation to neck circumference (n = 1354)

NAFLD	Neck circumference				
	Highest tertile versus lowest tertile			Per 1 SD, as continuous variable	
	Reference	HR (95% CI)	*p* for trend	HR (95% CI)	*p* value
Model 1	1.00	2.16 (1.60–2.92)	< 0.001	1.44 (1.27–1.65)	< 0.001
Model 2	1.00	1.79 (1.31–2.46)	< 0.001	1.36 (1.21–1.58)	< 0.001
Model 3	1.00	1.66 (1.21–2.27)	< 0.001	1.30 (1.13–1.49)	< 0.001
Model 4	1.00	1.51 (1.17–2.09)	0.001	1.25 (1.12–1.44)	0.002
Model 5	1.00	1.42 (1.15–1.97)	0.004	1.22 (1.10–1.41)	0.006

Model 1 was adjusted for age, smoking status, physical activity, and educational attainment. Model 2 was further adjusted for BMI and waist circumference. Model 3 was further adjusted for HOMA-IR, CRP, and adiponectin. Model 4 was further adjusted for model 3 variables and fasting glucose, post-loading plasma glucose, systolic blood pressure, diastolic blood pressure. Model 5 was further adjusted for lipid profiles and liver enzymes

**Fig. 3.**
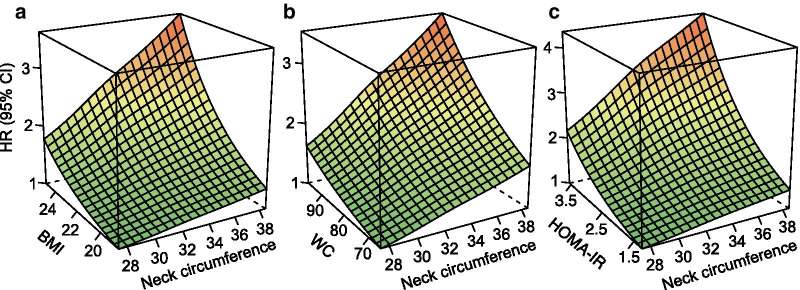
3-Dimensional nonalcoholic fatty liver diseases prediction plot. Prediction plot as a function of neck circumference levels (x-axis), BMI (y-axis) (**a**), waist circumference (y-axis) (**b**), and HOMA-IR (y-axis) (**c**) with the hazard ratio of incident nonalcoholic fatty liver diseases presented on the z-axis. BMI, body mass index; WC, waist circumference; HOMA-IR, homeostasis model assessment-insulin resistance

Considering the contribution of general obesity, abdominal obesity, and insulin resistance to the development of NAFLD, we further evaluated the combined effect of neck circumference with BMI, waist circumference, and HOMA-IR on fatty liver, respectively. The positive association between neck circumference and risk of incident NAFLD remained consistent across a wide range of BMI, waist circumference, and HOMA-IR (Fig. 3). And subjects with larger neck circumference combine with higher BMI, waist circumference, or HOMA-IR have a substantially increased incidence of NAFLD.

### Subgroup analyses

#### Stratified analyses of the associations between per 1-SD increment in neck circumference and nonalcoholic fatty liver disease

**Fig. 4:**
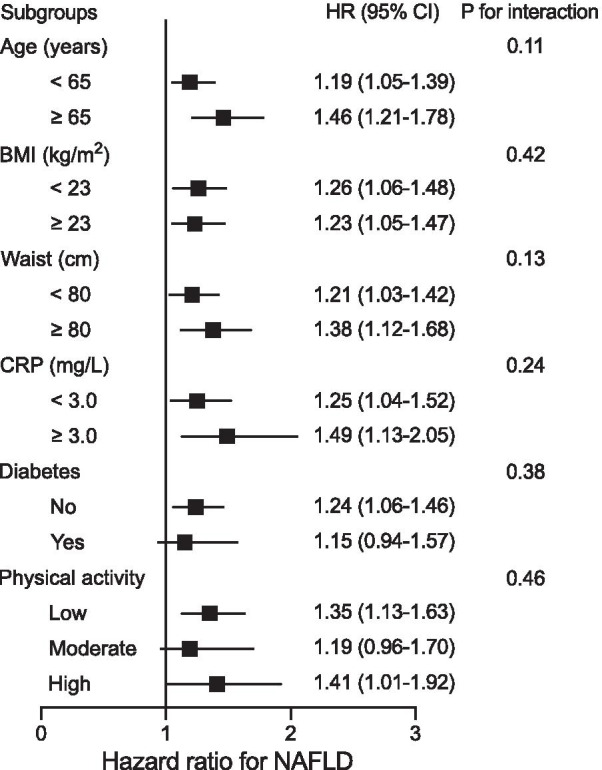
Stratified analyses of the associations between per 1-SD increment in neck circumference and nonalcoholic fatty liver disease. Model was adjusted for age, smoking status, physical activity, educational attainment, BMI, waist circumference, HOMA-IR, CRP, adiponectin, fasting plasma glucose, post-loading plasma glucose, systolic blood pressure, diastolic blood pressure, lipid profiles, and liver enzymes

Subgroup analyses were performed to examine potential effect modifiers, stratified by age, BMI, waist circumference, CRP, diabetes status, and physical activity. In the stratified analyses, the positive associations between per 1-SD increment in neck circumference and the risk of incident NAFLD remained consistent across all subgroups (Fig. 4). No interaction was observed with any of the variables (all p for interaction > 0.1).

## Discussion

In the present study, we observed that relatively large neck circumference was associated with the prevalence and incidence of NAFLD among postmenopausal women with normal BMI. Higher neck fat accumulation was also associated with metabolic dysregulation and inflammation characterized by insulin resistance, elevated triglycerides, and CRP. Collectively, these findings indicate the importance of excessive neck fat accumulation to predict the development of NAFLD among postmenopausal women with normal BMI.

Epidemiological studies have shown that sex and menopause affect the prevalence and incidence of NAFLD [14]. In women, the relations between visceral fat accumulation and metabolic profile were greater than in men [15]. Furthermore, as age increases and estrogen levels decrease, women after menopause are prone to insulin resistance, hyperlipidemia, and visceral fat accumulation [16, 17], all of which are known risk factors for NAFLD. The robust protective role of estrogens was revealed in epidemiological studies, where the prevalence of NAFLD is lower in premenopausal women compared to men or postmenopausal women [18]. As the main circulating estrogen, 17β-estradiol has effects beyond reproductive health. In insulin-sensitive tissues like skeletal muscle, 17β-estradiol receptor α has a positive effect on insulin signaling and glucose transporter-4 expression [19], which indicates that 17β-estradiol may directly regulate insulin action. Meanwhile, another study showed that exposure to 17β-estradiol restores insulin sensitivity and glucose tolerance in high-fat diet-fed ovariectomized mice [20]. In addition to insulin sensitivity, estrogens can also affect adipose tissue metabolism, energy expenditure, and hepatic glucogenesis [21]. Collectively, the protective effect of estrogen on NAFLD is a combination of multiple factors and its underlying mechanism remains to be further studied.

Previous researches reported that higher BMI, waist circumference, HOMA-IR, elevated triglyceride levels, and reduced HDL-cholesterol levels were associated with NAFLD among non-obese individuals [22]. In our study, neck circumference was significantly correlated with BMI, waist circumference, HOMA-IR, and triglycerides, and negatively correlated with HDL-C, consistent with previous studies [7, 23]. Moreover, the positive association between neck circumference and incidence of NAFLD remained consistent across a wide range of BMI, waist circumference, and HOMA-IR among postmenopausal women with normal weight. Additionally, previous cross-sectional studies have shown that neck circumference was an independent predictor for NAFLD in the general population [24] and non-obese men [25]. Our strong evidence indicating the relation of neck circumference and incidence of NAFLD even in postmenopausal women with normal BMI may validate their results and provide valuable clues for further studies.

It is well established that obesity is a heterogeneous disorder. BMI is a common screening measure to identify subjects with abnormal body fat distribution. However, BMI cannot provide accurate information about the regional distribution of body fat. Moreover, subjects within the same BMI could have considerable differences in the amount and distribution of regional fat accumulation. Previous studies have demonstrated that differences in body fat distribution result in specific metabolic complications [26, 27]. Thus, neck circumference, a proxy of neck subcutaneous fat, began to show diagnostic value for evaluating metabolic disturbances.

As an alternative measurement of upper-body subcutaneous fat, neck circumference is a great indicator of ectopic fat distribution. Subcutaneous fat in the upper body accounts for a much greater proportion of systemic free fatty acids released and is more lipolytically active than lower body adipose tissue [7]. Large neck circumference means excessive accumulation of subcutaneous fat in the neck, which contributes to a greater flux of the free fatty acid released into the circulation. Subsequently, elevated free fatty acid contributes to increased synthesis and ectopic deposition of triglycerides, insulin resistance, and inflammation [28]. In addition, increased free fatty acids are involved in impaired glucose homeostasis by inhibiting glucose uptake, oxidation, glycogen synthesis, and increasing output hepatic glucose [29]. Concomitantly, excessive free fatty acids could trigger oxidative stress, an early instigator of NAFLD [30], and endoplasmic reticulum stress which intersects with various inflammatory and stress signaling pathways through unfolded protein response [31]. The excessive free fatty acids release derived from neck subcutaneous adipose might be a potential link between neck circumference and NAFLD.

In our study, neck circumference was positively correlated with C-reactive protein, and negatively correlated with adiponectin. As we best known, the abnormal accumulation of fat is associated with adipose tissue metabolic capacities, endocrine, and immune function, which along with altered lipid mediators, adipokines, pro- or anti-inflammatory cytokines, and impaired signaling pathways that are involved in metabolic abnormalities [32]. In addition to being a depot of fat, the adipose tissue is also a highly active endocrine organ, secreting various biologically active molecules, collectively termed adipokines [33]. When adipose tissue expands, the capacity of adipocytes to act as endocrine cells and secrete a variety of adipokines is altered in subjects with NAFLD [34]. These altered kinds and levels of adipokine are associated with dysregulation of triglyceride, fatty acids metabolism, and insulin resistance [35]. Moreover, due to excessive fat accumulation and substantial infiltration of immune cells, a specific crown-like disposition of macrophages around single necrotic adipocytes occurs in subjects with NAFLD [36]. Subsequently, proinflammatory pathways were activated, and a variety of proinflammatory cytokines and chemokines were overflowed that contribute to low-grade inflammation and insulin resistance [32]. In general, adipose dysfunctions, inflammation, and stress partly linking neck obesity to insulin resistance and NAFLD.

Several potential limitations of the current study should also be noted. First, we did not quantify the tissue composition of the neck. The use of neck circumference to assess the neck subcutaneous fat was a convenient and practical way but was unable to quantify the fat accumulation and muscle mass. Hence, the amount and size of subcutaneous adipocyte and muscle fat are unclear. Second, due to ultrasonographic examination was performed to determine the presence of NAFLD, the sensitivity of liver ultrasonography may vary depending on the hepatic fat content. Nevertheless, when performed properly, ultrasonography has been reported to detect as little as ≥ 5% hepatic fat content. Although as discussed above, liver ultrasonography offers several strengths including the non-invasive nature of the test, portability, low cost, and simplicity of use, make it further applicable and acceptable in large-scale epidemiological studies, particularly in developing countries. Third, given the diagnosis of NAFLD was based on ultrasound imaging, NAFLD patients in our study were in at least a moderate stage of the disease. Therefore, in the present study, we were unable to determine the relationship between neck circumference and mild-stage NAFLD. Fourth, we did not assess the impact of excluded data on the relationship between neck circumference and incident NAFLD.

## Conclusions

Large neck circumference was significantly associated with an increased risk of NAFLD among postmenopausal women with normal BMI. Measurement of neck circumference may provide a more complete understanding of NAFLD risk associated with variation in fat distribution among postmenopausal women with normal BMI.

## Supplementary Information

**Additional file 1: Supplementary Table S1**. Baseline characteristics of study participants.

## Data Availability

The datasets generated during and/or analyzed during the current study are not publicly available but are available from the corresponding author on reasonable request.

## Abbreviations

ALT: Alanine aminotransferase
AST: Aspartate transaminase
BMI: Body mass index
CRP: C-reactive protein
DBP: Diastolic blood pressure
FPG: Fasting plasma glucose
GGT: γ-Glutamyltransferase
HbA_1c_: Glycated hemoglobin
HDL-C: High-density lipoprotein cholesterol
HOMA-IR: Homeostasis model assessment-insulin resistance
LDL-C: Low-density lipoprotein cholesterol
NAFLD: Nonalcoholic fatty liver disease
PPG: Postprandial plasma glucose
SBP: Systolic blood pressure
WC: Waist circumference
