# Supplementary material for: Neck circumference as an independent predictor for NAFLD among postmenopausal women with normal body mass index
Source: Nutr Metab (Lond). 2021 Mar 17;18:30. doi: 10.1186/s12986-021-00562-3 (PMC7967966; doi:10.1186/s12986-021-00562-3)
Supplement: Supplementary file 1 — Additional file 1: Supplementary Table S1. Baseline characteristics of study participants. [file 12986_2021_562_MOESM1_ESM.docx]

Supplementary data

Supplementary Table S1. Baseline characteristics of study participants.

| Variables | Remaining at year 3.1 follow-up  (n = 1,354) | Lost to follow-up  (n = 209) | p value |
| --- | --- | --- | --- |
| Age (years) | 58.6 ± 5.6 | 59.1 ± 5.7 | 0.23 |
| Current smoker, n (%) | 69 (5.0) | 11 (5.3) | 0.92 |
| Educational attainment, n (%) |  |  | 0.93 |
| 0-6 | 355 (26.2) | 54 (25.8) |  |
| 7-9 | 620 (45.8) | 97 (46.4) |  |
| ≥10 | 379 (28.0) | 58 (27.8) |  |
| Physical activity, n (%) |  |  | 0.89 |
| Low | 980 (72.4) | 151 (72.2) |  |
| Moderate | 279 (20.6) | 45 (21.5) |  |
| High | 95 (7.0) | 13 (6.2) |  |
| BMI (kg/m^2^) | 22.22 ± 1.62 | 22.21 ± 1.56 | 0.93 |
| Waist circumference (cm) | 78.1 ± 7.3 | 78.5 ± 6.3 | 0.45 |
| SBP (mmHg) | 127.72 ± 18.02 | 127.92 ± 18.50 | 0.88 |
| DBP (mmHg) | 77.32 ± 9.65 | 76.59 ± 9.96 | 0.31 |
| FPG (mmol/L) | 6.00 ± 1.37 | 5.98 ± 1.33 | 0.84 |
| PPG (mmol/L) | 7.94 ± 3.20 | 7.99 ± 3.43 | 0.83 |
| HbA_1c_ (%) | 5.87 ± 0.83 | 5.97 ± 0.86 | 0.11 |
| HOMA-IR | 1.48 (1.17-1.96) | 1.50 (1.14-1.86) | 0.56 |
| HDL-C (mmol/L) | 1.33 ± 0.33 | 1.30 ± 0.30 | 0.22 |
| LDL-C (mmol/L) | 2.68 ± 0.76 | 2.62 ± 0.77 | 0.29 |
| Total cholesterol (mmol/L) | 4.77 ± 1.00 | 4.74 ± 0.98 | 0.69 |
| Triglycerides (mmol/L) | 1.20 (0.90-1.61) | 1.18 (0.85-1.55) | 0.46 |
| ALT (U/L) | 12 (9-16) | 11 (8-15) | 0.09 |
| AST (U/L) | 19 (15-23) | 18 (15-22) | 0.13 |
| GGT (U/L) | 15 (11-21) | 14 (11-19) | 0.08 |
| CRP (mg/L) | 1.09 (0.58-2.01) | 1.01 (0.62-1.93) | 0.52 |
| Adiponectin (mg/L) | 3.81 ± 1.17 | 3.76 ± 1.15 | 0.56 |
| Neck circumference (cm) | 31.9 ± 2.4 | 32.1 ± 2.4 | 0.26 |

Abbreviations: ALT, alanine aminotransferase; AST, aspartate transaminase; BMI, body mass index; CRP, C-reactive protein; DBP, diastolic blood pressure; FPG, fasting plasma glucose; GGT, γ-glutamyltransferase; HbA_1c_, glycated hemoglobin; HDL-C, high-density lipoprotein cholesterol; HOMA-IR, homeostasis model assessment-insulin resistance; LDL-C, low-density lipoprotein cholesterol; PPG, postprandial plasma glucose; SBP, systolic blood pressure.
